# Reproducibility in Nerve Morphometry: Comparison between Methods and among Observers

**DOI:** 10.1155/2013/682849

**Published:** 2013-06-13

**Authors:** Antônio Paulo da Costa Bilego Neto, Fernando Braga Cassiano Silveira, Greice Anne Rodrigues da Silva, Luciana Sayuri Sanada, Valéria Paula Sassoli Fazan

**Affiliations:** ^1^Department of Neurosciences and Behavioral Neurosciences, School of Medicine of Ribeirão Preto, University of São Paulo, 14049-900 Ribeirão Preto, SP, Brazil; ^2^Department of Surgery and Anatomy, School of Medicine of Ribeirão Preto, University of São Paulo, Avenida Bandeirantes 3900, 14049-900 Ribeirão Preto, SP, Brazil

## Abstract

We investigated the reproducibility of a semiautomated method (computerized with manual intervention) for nerve morphometry (counting and measuring myelinated fibers) between three observers with different levels of expertise and experience with the method. Comparisons between automatic (fully computerized) and semiautomated morphometric methods performed by the same computer software using the same nerve images were also performed. Sural nerves of normal adult rats were used. Automatic and semiautomated morphometry of the myelinated fibers were made through the computer software KS-400. Semiautomated morphometry was conducted by three independent observers on the same images, using the semiautomated method. Automatic morphometry overestimated the myelin sheath area, thus overestimating the myelinated fiber size and underestimating the axon size. Fiber distributions overestimation was of 0.5 **μ**m. For the semiautomated morphometry, no differences were found between observers for myelinated fiber and axon size distributions. Overestimation of the myelin sheath size of normal fibers by the fully automatic method might have an impact when morphometry is used for diagnostic purposes. We suggest that not only semiautomated morphometry results can be compared between different centers in clinical trials but it can also be performed by more than one investigator in one single experiment, being a reliable and reproducible method.

## 1. Introduction

Morphometry enables us to describe structures in quantitative terms and in particular reveals minimal morphological differences between states of function. In pathology application, in the field of diagnostic/prognostic, the need for objective methods is evident [1], and this can be achieved by morphometry. There is no doubt that morphometry of peripheral nerves is relevant for a complete quantitative description of structure, size, and components of the endoneurial space and of changes as they occur in morphogenesis or in pathology. Morphometry of myelinated fibers has been found to be increasingly useful in studies of development, aging, regeneration, neurotoxicity, and various pathologic conditions [2], such as diabetes and hypertension. Fiber diameter is used as the basis for classification of nerve fiber populations and has been correlated with conduction velocity.

Despite its evident relevance, peripheral nerve morphometry is still a controversial issue. Most authors that use this technique on a routine basis either for diagnostics purpose or for neuropathy research do not agree on which would be the most reliable method for morphometry, manual or automatic. There is no doubt that manual morphometry is prohibitively time consuming, difficult to perform correctly, tedious, predisposing to observer fatigue, and subject to many sources of error [3–6]. On the other hand, fully automatic computerized image analysis systems, despite being described as precise and far less time consuming [3, 7], sometimes require specialized equipment, top-of-the-line computers with large and expensive memory, and sophisticated software, being an expensive technique and sometimes difficult to implement [6, 8]. Also, fully automatic morphometry is often associated to conversion of uncorrected error in image interpretation to spurious data [2, 8]. Thus, it seems that the methods that permit user interaction, termed semiautomated, are the current gold standard for nerve morphometry. Nevertheless, few reports deal with the reproducibility between methods, and the question if one would be able to compare results obtained with two different methods remains unanswered.

Another important question that demands immediate attention on nerve morphometry is that it is not clear if results obtained by different investigations using the same interactive method are comparable. Even more urgent is the understanding if a single morphometry method that requires any kind of interference by the investigators is reliable and comparable. This need has become more evident since a large number of quantitative nerve studies are now available in the literature, but the reproducibility of the results is questionable. Thus, we aimed to investigate the reproducibility of a semiautomated method for nerve morphometry between three observers with different levels of expertise and experience with the method. We also compared the results obtained with the same computer software but used in two different settings: automated and semiautomated.

## 2. Methods

Experiments were performed on 90-day-old female Wistar rats (*N* = 6), from the School of Medicine of Ribeirão Preto animal care facility. Animals were born and raised in a carefully regulated environment maintained at 21 to 23°C, 40 to 70% relative humidity, and 12/12 hour light/dark cycle and received tap water and normal rat chow *ad libitum*. All procedures adhered to “*The ARRIVE guidelines: Animal Research: Reporting In Vivo Experiments, originally published in PLoS Biology, June 2010*” and were approved by the Institutional Ethics Committee for Animal Research (CETEA-Comitê de Ética em Experimentacão Animal, protocol number 184/2005). Every effort was made to minimize the suffering of the animals and the number of animals used. 

**Figure 1:**
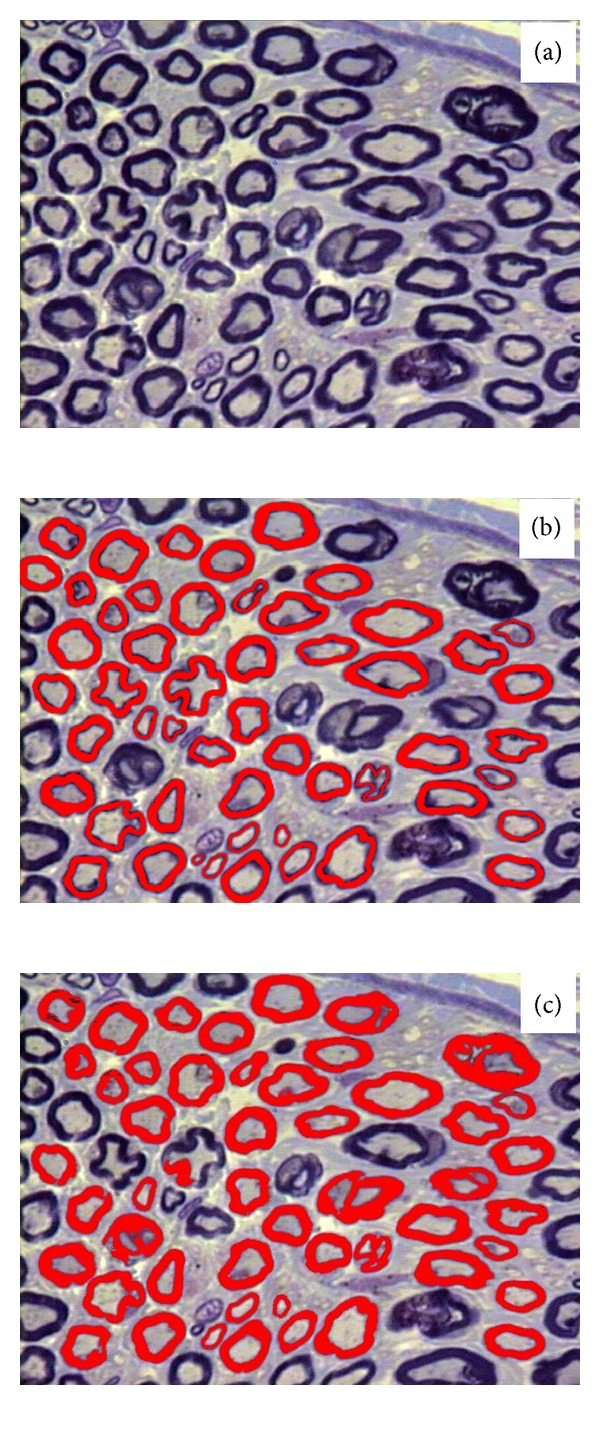
Representative semithin cross-sections of an endoneurial area of the sural nerve digitized for morphometry (a). The same area is shown in (b) and (c), with the myelin sheaths marked in red (fiber mask) by the semiautomated (b) and automatic (c) methods. Note the “misdetection” of irregular fibers and artifacts by the automatic segmentation and the detection of the myelin sheath background pixels, even to the point of uniting two separate fibers. Toluidine blue stained, bars = 10 *μ*m.

The animals were anesthetized with sodium pentobarbital (Nembutal, 40 mg/kg, IP) and perfused through the left ventricle first with a phosphate buffered saline 0.05 M (pH 7.4) solution, followed by a 2.5% glutaraldehyde solution, in 0.1 M cacodylate buffer (pH 7.2). Both right and left sural nerves, from their origin in the hip (5 to 7 mm distal to the greater trochanter) through their distal branching at the lateral malleolus level, were carefully dissected without stretching, removed in one piece, and placed in the fixative solution for an additional 12 hours. Proximal and distal segments of the nerves were postfixed in 1% OsO_4_ in cacodylate buffer solution and dehydrated in graded ethanol. After epoxy resin embedding, nerves were cut transversally (0.3 *μ*m thick sections), stained with 1% toluidine blue solution, and observed under the oil immersion lens of a light microscope. The procedures for epoxy resin embedding and light microscopy were performed as described elsewhere [9, 10]. All nerve samples were histologically processed simultaneously in order to avoid any kind of bias introduced by the tissue preparation. For the myelinated fiber study, the endoneurial space of each nerve fascicle was fully scanned without overlap of the microscopic fields, with the aid of a microscope automatic motor plate. This scanning generated a number from 8 to 15 microscopic fields of 640 × 470 pixels, which were used to count and measure the myelinated fibers and respective axons. Fibers at the superior and left edges of the microscopic fields were counted while fibers at inferior and right edges were not counted, in order to avoid counting the same fiber twice. All myelinated fibers present in the endoneurial space were counted. Automatic or semiautomated morphometry was performed with the aid of the KS-400 (Kontron 2.0, Eching Bei Munchen, Germany) computer software. For the automatic procedure the software performed an automatic contrast enhancement and threshold adjustment of the myelinated fibers. Fiber segmentation was conducted as a function of pixel color and brightness. The system automatically recognized the myelin sheaths that were first marked in red color (fiber mask) (Figure 1) before generating a binary image of the myelin sheath. The inversion of these images generated the axons image (axon mask). Afterwards, the inner and outer diameters were automatically measured on the axon binary mask and on the fiber binary mask, respectively. The variables measured were fiber and axon area and minimum diameter, myelin sheath area, and *G* ratio (ratio between axon diameter and total fiber diameter; a measurement of the degree of myelination) [11, 12]. Histograms of fiber and axon size distributions were constructed with class intervals of 0.5 *μ*m. Morphometric data was stored on an IBM-PC hard drive for statistical analysis.

Semiautomated morphometry was performed on the proximal right segment of the sural nerves. Three independent observers used the same software and same microscopic images used for the automatic morphometry. Observer 1 was skilled with the method and used to perform sural nerve morphometry, thus presenting high knowledge of this nerve morphology and morphometry. Observer 2 was also skilled with the semiautomated morphometry method but not used to the sural nerve morphology. Observer 3 was under training with both: the semiautomated morphometry method and the sural nerve morphology and morphometry. The semiautomated morphometry consisted in the identification of the myelinated fibers by the observers and manual tracing of the myelin sheath of each intact fiber identified on the images (Figure 1). This tracing was performed with the aid of the Bamboo Connect Pen Tablet (Wacom Technology Corporation) system, which consists of a tablet with an active measuring area of 14.7 × 9.1 cm (resolution of 0.05 mm, digitizer speed of 133 points/sec, and a resolution of 1000 lines/cm) and a pen (or cursor) for coordinate selection, connected to the computer through the USB type A plug. Obtained data was stored on an IBM-PC hard drive for further analysis.

Morphometric data were tested for normal distribution by the Kolmogorov-Smirnov test. When parameters in the same side (proximal versus distal segments) presented a normal distribution, they were compared by the paired *t*-test. Otherwise, they were compared by the nonparametric test of Wilcoxon for paired samples. Right and left segments from the same level (proximal or distal) were tested by the unpaired Student's *t*-test for normally distributed data. Otherwise, comparisons were made by the nonparametric test of Mann-Whitney. These tests were used on data obtained by the skilled observer (observer 1) for each of the methods (automatic or semiautomated) and also on the comparisons between automatic versus semiautomated morphometry. Data obtained by the three observers (average values) on the proximal right segments were compared by one-way analysis of variance (ANOVA) followed by the *post hoc* test of Holm-Sidak. Differences were considered significant if *P* < 0.05. Data are presented as mean ± standard error of mean (SEM).

## 3. Results

### 3.1. Comparison between Methods

Myelinated fibers values, obtained either by the automatic or the semiautomated methods, were compared. Proximal and distal segments of the sural nerves from both sides showed no differences with both methods, being the nerves longitudinally symmetric. Also, the comparison between segments of the same level between sides did not show differences with either of the methods, being the nerves also laterally symmetric.

**Figure 2:**
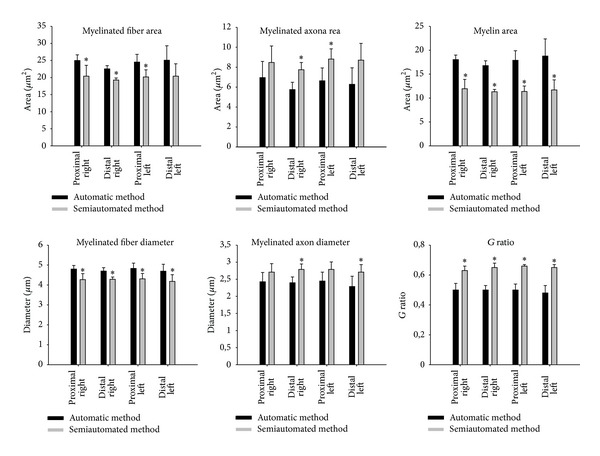
Comparison between the average morphometric parameters of the myelinated fibers and respective axons from the sural nerves of young (90-day-old) female Wistar rats, obtained by the automatic and semiautomated methods. Data presented as average ± standard error of mean. * indicates significant difference compared to automatic method (nonparametric test of Mann-Whitney for *G* ratio values and unpaired Student's *t*-test for fiber, axon and myelin sheath area, and fiber and axon diameter).

**Figure 3:**
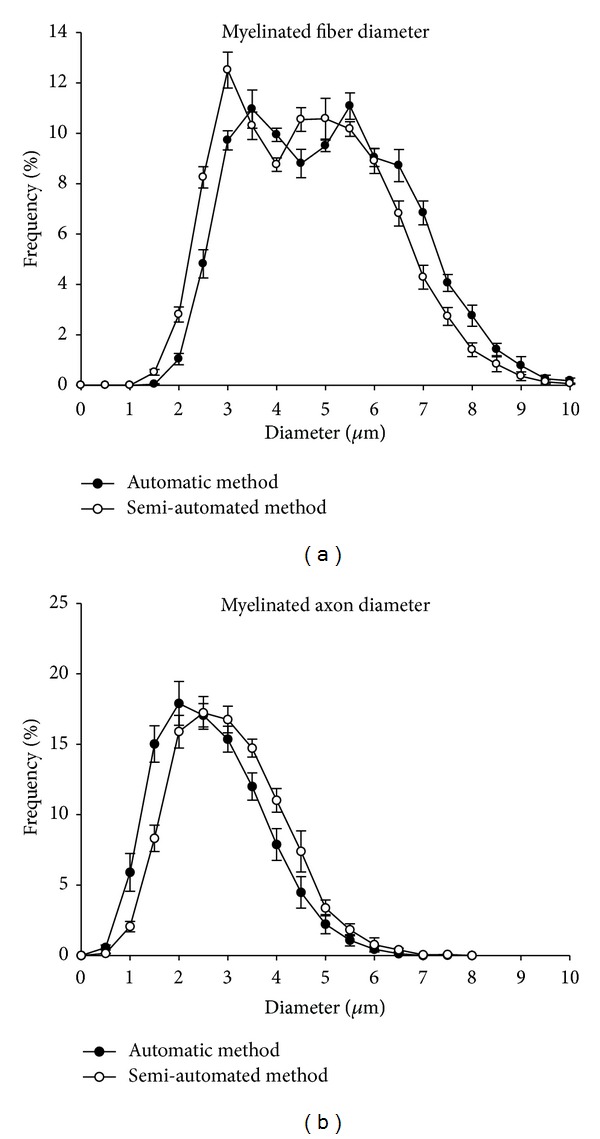
Size distribution of myelinated fibers (a) and respective axons (b) of the proximal segment of the right sural nerve of young (90-day-old) female Wistar rats, obtained by automatic (black circles) and semiautomated (white circles) morphometry. Note that myelinated fibers distributions are bimodal while axon distributions are unimodal. Note also that the fiber distribution obtained with the automatic method is skewed to the right in 0.5 *μ*m while the axon distribution is skewed to the left in 0.5 *μ*m, due to the overestimation of the myelin sheath thickness.

Figure 2 shows the average values of the myelinated fibers morphometry obtained with the automatic and semiautomated methods. Myelinated fiber size (area and diameter) as the myelin sheath area was generally larger with the automatic method, while myelinated axon size (area and diameter) and the *G* ratio were larger with the semiautomated method. The most obvious difference was observed on the myelin sheath area value that had a direct impact on the *G* ratio values. The myelinated fibers and respective axon diameter distributions were not different between segments and sides when tested with the same method. Nevertheless, the comparison between the fiber distributions obtained with both methods shows a 0.5 *μ*m shift to the right with the automatic method, in all class intervals (Figure 3), while myelinated axon distributions are shifted to the left with the automatic method (Figure 3). 

### 3.2. Comparison between Observers

**Figure 4:**
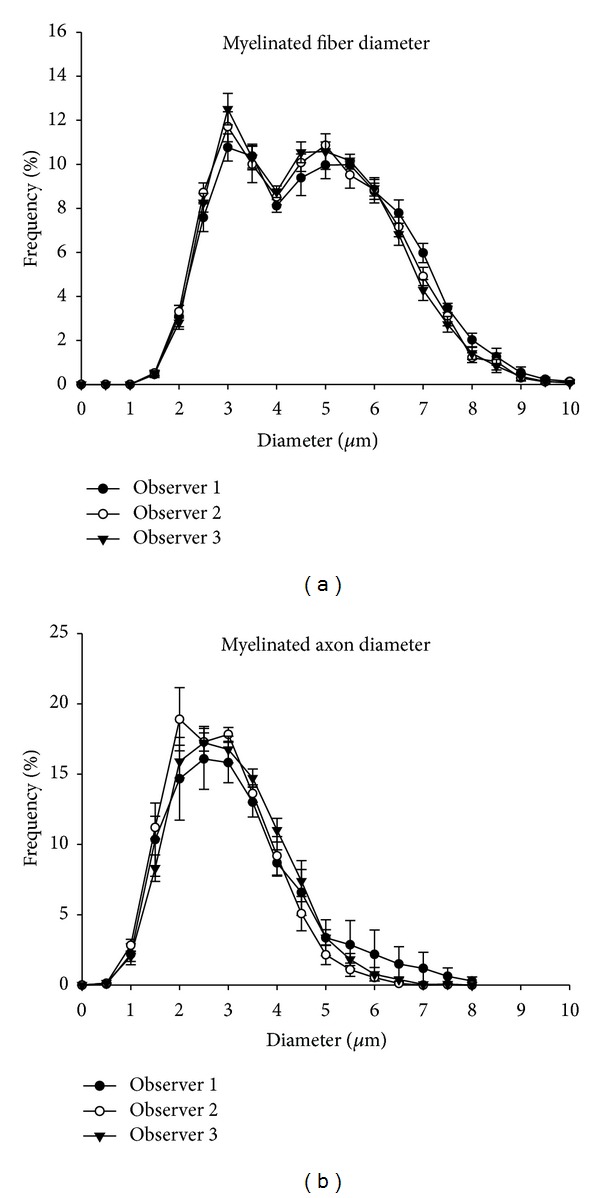
Size distribution of myelinated fibers (a) and respective axons (b) of the proximal segment of the right sural nerve of young (90-days-old) female Wistar rats, obtained by three different observers, using the semiautomated method. Note that myelinated fibers distributions are bimodal while axon distributions are unimodal, with a complete superposition of the distributions obtained by the observers.

The average values for the myelinated fibers and respective axons size (area and diameter), the myelin sheath area, the *G* ratio, and the number of fibers counted and measured by the three independent observers, using the semiautomated method, are shown in Table 1. A difference on the myelin area average was detected between the three observers, but the *G* ratio was different only between observers 1 and 3. No other differences were found.  Figure 4 shows the myelinated fibers and respective axon diameter distributions obtained by the three observers. No differences between observers were found on the histograms shape, peaks, or class intervals on both histograms. 

## 4. Discussion

The present study shows first that the fully automatic method for semithin sectioned myelinated fiber morphometry overestimates the myelin sheath size of normal fibers, thus reducing the *G* ratio, and second that the semiautomated morphometry is reproducible between observers.

The possibility of overestimation of the myelin sheath thickness by an automatic morphometry method was mentioned by Campadelli et al. [13]. The authors discuss that automatic methods that take into consideration the edge detection produce imprecise segmentation leading to fiber loss or alteration of their contour. More important is that “background pixels” of the myelin sheath edges can be detected; that would falsely increase the myelin thickness, leading to an increase in total fiber size and a decrease in the axon size. Our morphometric results (Figure 2) are in accordance to the hypothesis of background pixels detection, since we observed that myelinated fibers area and diameter are significantly larger with the automatic morphometry, while the axon area and diameter are significantly smaller with this method, when compared to the semiautomated one. When looking carefully to Figure 1, despite the “misdetection” of irregular fibers and artifacts by the automatic segmentation, we can easily see the detection of the myelin sheath background pixels, even to the point of uniting two separate fibers. The most common errors in automatic nerve morphometry [8] were also detected in the present study, that are (1) interpretation by the computer program of close related structures as single ones, (2) recognition and measurement of artifacts (false positive), and (3) missed detection of true axons (false negative), particularly the small ones. Urso-Baiarda and Grobbelaar [8] also discussed that “excessive stain” on fibers boundaries can easily be maldetected by automatic morphometry. In this regard, it is important to take into consideration the *G* ratio values compared between the two methods investigated in the current study. 

The correlation between the myelin sheath and the diameter of the respective axon is known since 1905 [14] and may differ significantly between nerves and also between large and small fiber classes within individual nerves [15]. Rushton [11] suggested that *G* ratio values between 0.6 and 0.7 would be the theoretically optimal for the spread of current from one node of Ranvier to the next, being the best values for the maximum conduction velocity of a myelinated fiber. Since we studied normal nerves, we would expect most of the fibers to show a *G* ratio between 0.6 and 0.7. Nevertheless, we observed reduced (smaller than expected) average *G* ratio with the automatic morphometry (values close to 0.5) while with the semiautomated method these values were closer to the ones expected for normal fibers. The task of morphometry is to discover biological regularities and point out their dependence. Every deviation from normality can indicate developmental tendencies or reveal pathological processes [16]. The exact determination of myelin sheath-axon ratios is especially essential when interpreting pathological processes in peripheral nerves [17], and neuropathies are currently diagnosed on the basis of clinical information integrated with the morphometric analysis of nerve specimens [13]. Accuracy in the measurements is mandatory in order to allow a correct interpretation of data. In comparing the *G* ratio data between automatic and semiautomated methods, associated with the interpretation of the myelinated axon distribution, one could interpret the results as the presence of axonal atrophy in normal nerves. In this way, despite the time and effort that the semiautomated morphometry might demand, we still believe that the interference of an observer in nerve morphometry is crucial.

On the other hand, every time we have the interference of an observer, the question of reproducibility of the method comes up. Since nerve morphometry became more accessible, the number of studies on the normal pattern of development, postnatal maturation, and aging of nerves increased considerably, not only in humans [18–21] but also in experimental animals [9, 10, 22–24]. Since the spatial distribution of the myelinated fibers are not uniform in human [25, 26] and animal nerves [27, 28] and pathological changes may be focal, multifocal, or diffuse in abnormal nerves, sampling methods for nerve morphometry have been investigated [26, 29], still with controversial conclusions. An investigation of the correlation between morphometric data and functional recovery [30] showed that, in crush injury, 71% of the motor fibers are correctly directed to the muscle after two months. Interestingly, clinical recovery was practically complete at 4 weeks. Despite that there was a discrete increase in fiber number (26%), their diameter and myelin sheath thickness were reduced in 38%. These results suggest that a nerve is able to function correctly even if it has regenerated only 70% of its fibers. Regarding the reproducibility of the morphometric assessment of nerves, Sima et al. [31] suggested that quantitative morphometry could be used in clinical trials after a formal evaluation of the within-subject and between subject variability of the morphometric measures. The authors investigated nerves from diabetic patients and the morphometric elements of nerve damage in this pathological condition, concluding that the investigation of fiber loss, atrophy, and injury was reproducible. In the present study, we investigated the most useful morphometric parameters of normal myelinated fibers and respective axons, not only the average numbers but also the fiber and axons size distribution, showing no differences between observers with different skills. 

## 5. Conclusions

 The possibility of overestimation of the myelin sheath thickness of normal fibers by an automatic morphometry method should be considered particularly when using this method for diagnostic purposes.

Also, we suggest that not only semiautomated morphometry results can be compared between different centers in clinical trials but it can also be performed by more than one investigator in one single experiment, being a reliable and reproducible method.

## Figures and Tables

**Table 1:** Average morphometric parameters of the myelinated fibers from the proximal segments of the sural nerve of young (90-day-old) female Wistar rats, obtained by three different observers, using the semiautomated method.

	Observer 1	Observer 2	Observer 3
MF area (*µ*m^2^)	23.3 ± 0.7	21.5 ± 0.5	21.4 ± 0.7
MF diameter (*µ*m)	4.6 ± 0.1	4.4 ± 0.1	4.4 ± 0.1
AX area (*µ*m^2^)	7.4 ± 0.6	7.8 ± 0.6	8.9 ± 0.6
AX diameter (*µ*m)	2.5 ± 0.1	2.6 ± 0.1	2.8 ± 0.1
Myelin area (*µ*m^2^)	15.9 ± 0.2^#†^	13.7 ± 0.4^∗†^	12.5 ± 0.4^∗#^
G ratio	0.6 ± 0.0	0.6 ± 0.0	0.6 ± 0.0*
MF counted	1058 ± 42	975 ± 53	1055 ± 42
MF measured	873 ± 35	838 ± 42	838 ± 41

MF: myelinated fiber; AX: myelinated axon. *indicates significant difference compared to observer 1; ^#^indicates significant difference compared to observer 2; ^†^indicates significant difference compared to observer 3 (one-way analysis of variance followed by the *post hoc* test of Holm-Sidak).
